# Integrating Geospatial Data and Measures of Disability and Wealth to Assess Inequalities in an Eye Health Survey: An Example from the Indian Sunderbans

**DOI:** 10.3390/ijerph16234869

**Published:** 2019-12-03

**Authors:** Soumya Mohanty, Emma Jolley, RN Mohanty, Sandeep Buttan, Elena Schmidt

**Affiliations:** 1 Monitoring Evaluation Research and Learning, Sightsavers India 110020, India; msoumya@sightsavers.org; 2 Research, Sightsavers, Haywards Heath RH16 3BW, UK; 3 India Directorate Sightsavers India 110020, India; rnmohanty@sightsavers.org; 4 Programme Development and Innovation Team, Sightsavers India 110020, India; sbuttan@sightsavers.org; 5 Programme Development, Evidence and Research, Sightsavers, Haywards Heath RH16 3BW, UK; eschmidt@sightsavers.org

**Keywords:** Blindness, visual impairment, cataract surgical coverage, GIS and spatial analysis

## Abstract

The Sunderbans are a group of delta islands that straddle the border between India and Bangladesh. For people living on the Indian side, health services are scarce and the terrain makes access to what is available difficult. In 2018, the international non-governmental organisation Sightsavers and their partners conducted a population-based survey of visual impairment and coverage of cataract and spectacle services, supplemented with tools to measure equity in eye health by wealth, disability, and geographical location. Two-stage cluster sampling was undertaken to randomly select 3868 individuals aged 40+ years, of whom 3410 were examined. Results were calculated using standard statistical processes and geospatial approaches were used to visualise the data. The age–sex adjusted prevalence of blindness was 0.8%, with higher prevalence among women (1.1%). Cataract Surgical Coverage for eyes at visual acuity (VA) 3/60 was 86.3%. The study did not find any association between visual impairment and wealth, however there were significant differences by additional (non-visual) disabilities at all levels of visual impairment. Geospatial mapping highlighted blocks where higher prevalence of visual impairment was identified. Integrating additional tools in population-based surveys is critical for measuring eye health inequalities and identifying population groups and locations that are at risk of being left behind.

## 1. Introduction

Visual impairment is a global public health problem. Estimates from the World Health Organisation (WHO) suggest that 253 million people suffer from visual impairment globally, of whom 36 million are blind [1]. Worldwide, more than 75% of visual impairment is avoidable, which means it can be prevented or treated with access to good quality services, however this access is far from universal. Around 89% of visually impaired people live in low- and middle-income countries (LMICs) and 55% are women, indicating inequalities in the availability and access to eye care. Cataract remains the leading cause of blindness and the second leading cause of severe and moderate visual impairment in LMICs [2].

The WHO’s Universal Eye Health Coverage: Global Action Plan (GAP) 2014–2019 calls for a 25% reduction in visual impairment [3] and recommends that Member states undertake regular population based surveys to monitor this goal and to support the delivery of eye health programmes.

There is an increasing understanding that both disease burden and access to health services vary across and between geographic areas as well as by individual and community-level characteristics. The use of geographic information system (GIS) data and data disaggregated by population characteristics reveals deeper insights into who is most disadvantaged and may require additional or targeted efforts to be reached. By utilising such data, programme managers and service providers can make faster and more effective decisions, maximising the efficiency of the limited resources that are available [3]. The past decade has seen a sharp rise in the number of epidemiological studies employing GIS and other data disaggregation methods to understand health inequalities and health-related behaviours. However, only a few studies explored these approaches in eye health related studies [3,4].

With financial support from Standard Chartered Bank under the “Seeing is Believing” initiative, the international non-governmental organisation (iNGO) Sightsavers worked with the local government and other partners to strengthen eye care services in the Indian Sundarbans, a group of delta islands that are located in the extreme south of West Bengal. In 2018, Sightsavers conducted a population-based eye health survey based on the standard RAAB methodology was supplemented by tools which measured equity of access to eye care services by wealth, disability, and geographic location. This article presents key findings of this survey and describes how the inclusion of geospatial data and measures of disability and wealth allows for a more nuanced understanding of the prevalence of visual impairment, cataract surgical coverage, and eye health inequalities.

## 2. Materials and Methods 

### 2.1. Study Area

**Figure 1:**
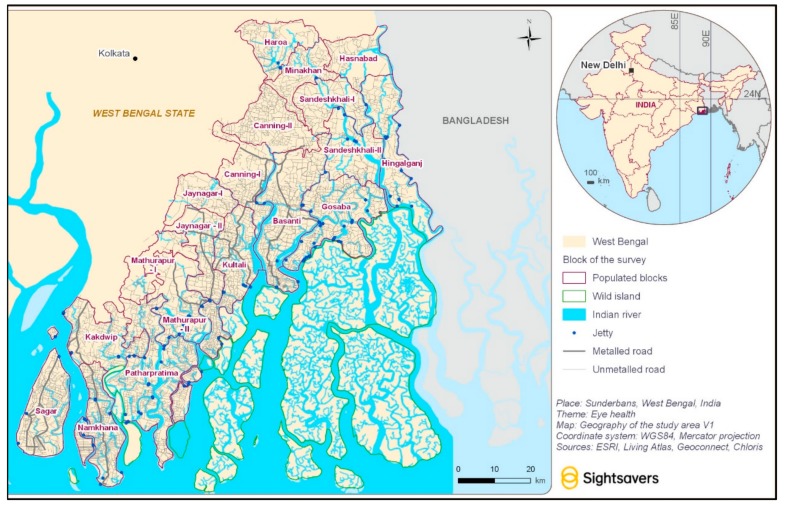
Study area: Sunderbans, India, 2018.

The Indian Sundarbans are comprised of 19 administrative blocks, which lie within two districts, the North and South 24 Parganas, and consist of villages that are spread over the islands and the adjacent mainland (Figure 1). The region is home to about 4.7 million people, almost half of whom (47%) belong to historically marginalised groups such as Scheduled Castes and Tribes [5]. About 29% of the population is above 40 years of age. People living in the Sunderbans face extreme geographical challenges, poverty, and difficulties regarding access to health services. These geographical challenges, however, vary across the blocks, with people living in the “remote” Sundarbans—the blocks that are adjacent to the forest area or the Bay of Bengal—facing much greater problems compared to those who live in the “peripheries” and closer to Kolkata.

### 2.2. Sampling and Sample Size

The survey of visual impairment was based on the standard RAAB methodology, with the eligible population including people aged 40+ years rather than 50+ years. The 2011 Census of India was used to inform the sampling frame [6]. The sample size that was required was 3782 people aged 40+ years. Two-stage cluster sampling was used to identify participants. Clusters (villages) were selected using probability proportional to size, and 50 eligible people in each cluster were selected using compact segment sampling [7]. In cases where the team came across a locked house, two attempts were made to revisit it.

### 2.3. Definitions and Tools

The survey followed the WHO visual impairment definitions (WHO—International Classification of Diseases 10) [8]. Blindness was defined as presenting visual acuity (VA) worse than 3/60 in the better eye, severe visual impairment (SVI) was defined as VA worse than 6/60 but better than or equal to 3/60 in the better eye, and moderate visual impairment (MVI) was defined as VA worse than 6/18 but better than or equal to 6/60 in the better eye. Cataract Surgical Coverage (CSC) was measured as the proportion of people who had been operated on for cataract out of those with operable cataract. Results are presented separately to show coverage with best corrected visual acuity (BCVA) of <3/60, <6/60, and <6/18 [9,10]. 

Visual acuity examination was conducted using the standard RAAB approach [11,12]. Visual acuity measurement was taken with available correction in daylight illumination for each eye using the Snellen Tumbling “E” letter with optotypes of sizes 12, 18, and 60 at 6 m, 3 m, and 1 m (if applicable). If the visual acuity with available correction was <6/12 in either eye, then the pinhole vision was measured following the same procedure to obtain a “best corrected” visual acuity. Presenting vision or presenting visual acuity (PVA) was defined by the visual acuity in the better eye using currently available correction, if any [13]. All participants underwent lens examinations in a darkened room. All eyes with available correction <6/12 were assigned a main cause for the visual impairment. The options were:

- Uncorrected refractive error: If best corrected vision equaled 6/12 or better;
- Unoperated cataract: if best corrected vision was worse than 6/12 and an obvious lens opacity was observed;
- Corneal scar: if best corrected vision was worse than 6/12 and obvious central corneal scarring was observed;
- Phthisis/globe abnormality: a shrunken or obviously damaged globe;
- Other: any other eye with best-corrected vision <6/12 not due to one of the above causes.

The current RAAB tool does not collect data on participant wealth or disability status. To collect these data, we integrated additional tools. Household relative wealth was measured using the India Equity Tool (IET), an asset-based tool, which allocates households to quintiles referenced against national level thresholds and shows whether your study participants are wealthier or poorer than the national population and allows disaggregation of data by wealth quintiles [14]. Data on household infrastructure (e.g., roof, floor, water source) and assets was collected from the heads of the households.

Disability was ascertained using the Washington Group Short Set of Questions of Disability (WGSS) [14] to measure limitations in activities. The WGSS measures functional difficulties in six domains (seeing, hearing, walking or climbing, remembering or concentrating, washing or dressing (self care), and communicating), with the answers on four point scale from no difficulty to cannot do at all. Disability was determined if an individual reported having a lot of difficulty or complete inability in at least one domain. A second indicator, non-visual disability, was also calculated if an individual reported having a lot of difficulty or complete inability in any domain other than seeing. Since this was the first time the WGSS was administered in the Sunderbans, the tool was cognitively tested in the local language i.e., Bengali, prior to the main survey among a small group of people in the Sunderbans region, which helped to finalise the translation of the tool into Bengali.

Data were collected using mobile tablets (Android based) and an app designed in CommCare [15]. GPS coordinates of each household were also collected as part of the survey.

### 2.4. Fieldwork Training and Procedures

The survey teams comprised of optometrists who received comprehensive training prior to the fieldwork for five days to ensure quality and strict adherence to the study protocol and ophthalmic examination procedures. The training included an overview of the study, survey tools, data collection procedures, tablet use, eye examination procedures, and ethics. The intra-observer variation (IOV) was tested to ensure a high degree of consistency between the teams.

### 2.5. Ethical Considerations

Ethics approval for the survey was obtained from the Institutional Review Board of Vivekananda Mission Ashram. The survey conformed to the principles of health research and all eligible participants were given information about the study and procedures in their local language. Informed written consent was obtained from all participants prior to the survey and examination. No financial incentives were provided for survey participants.

Following ophthalmic examination, all participants received feedback about their eyes and were advised to seek ophthalmic consultation if they had any concerns. Appropriate counselling was done after the examination and those who were identified with an eye problem were provided referral slips to visit the nearest partner hospital of Sightsavers in their local area.

### 2.6. Data Analysis 

Data was analysed using Stata [16] and maps were created using ArcGIS Pro 2.3.0 (ESRI, Redlands, CA, USA) [17]. Composite variables such as person visual acuity, cataract surgical coverage, disability, and wealth were calculated using recommended syntax and cut-offs. Age and sex adjusted estimates were calculated to account for differences in the sample and population structures. Descriptive statistics were generated to present the variables of interest; to examine the relationship between visual impairment and socio-economic characteristics, Chi-Squared test was conducted with a *p*-value of 0.05 considered the minimum acceptable to indicate a significant difference.

Spatial analysis at the block level was conducted using GIS mapping techniques. Analysis of clustering was conducted using a spatial autocorrelation function, which tested the hypothesis that visual impairment was randomly distributed. The administrative boundaries were the main base-maps used. ESRI and its living Atlas and Geoconnect were used as the major database sources [18]. ESRI provides a database on the main rivers, seas, and infrastructures and can be downloaded to prepare maps at small scales. The data were compared with the Census of India, 2011 tables to ensure that the administrative boundaries conformed to the last version that was used by the Government of India [19].

## 3. Results

### 3.1. Study Coverage

**Figure 2:**
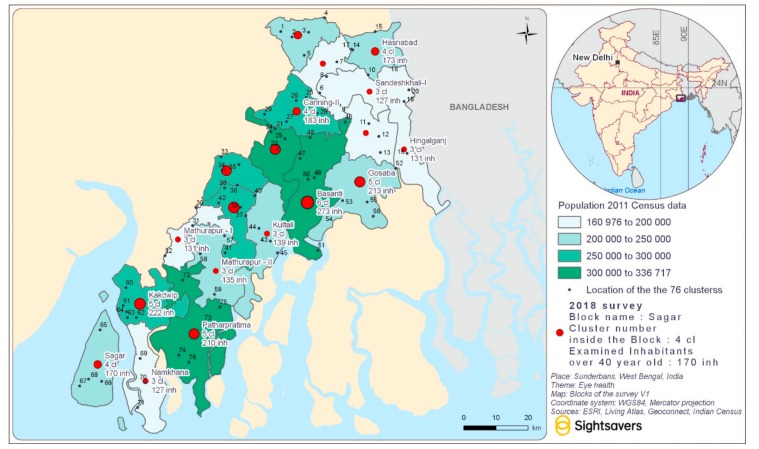
Blocks of Sunderbans, India included in the eye health survey.

Figure 2 shows the study area and describes the distribution of clusters and survey participants. Overall, 3868 individuals aged 40 or above were enumerated and 3410 were examined (response rate 88.2%). Around 50.8% of the enumerated participants were male, however men were more likely to be unavailable for an examination. This may be due to the geography of the Sunderbans as men often travel far for work.

### 3.2. Socio-Demographic Characteristics of Participants

In 1948 households with eligible participants, the median number of people aged 40+ years was 2. Over 70.5% of the households (n = 1373) were headed by men and 29.5% (n = 575) were headed by women. Over 45.7% of the households were Scheduled Caste (SC), 8.3% were Other Backward Caste (OBC), 3.6% were Scheduled Tribes (ST), and 42.5% were others (Non ST/SC/OBC). None of the surveyed households fell into the poorest quintile and only 12.9% fell into the second poorest quintile; the proportion of the households in the two wealthiest quintiles was 57.3%. Although generally such a distribution of participants across the quintiles indicates that study participants are relatively wealthier than the national population, in this survey, it is most likely due to the outdated national cut off points determined by the 2012 India Health and Demographic Survey (IHDS) II [20].

The mean age of the 3410 participants examined in the survey was 53 years. Nearly 40% of the participants were illiterate (cannot read or write), with much higher levels of illiteracy among women (47.8% compared to 26.8% among men). Around 37.5% of participants had primary education and 11.1% had secondary education (Table 1).

The prevalence of disability was 14.7% (16.5% among women and 12.9% among men). The prevalence of non-visual disability (excluding the seeing domain) was 11.1% (12.7% among women and 9.3% among men). The prevalence of disability increased with age from 4.3% among people aged 40–49 years to 70.8% among those aged 80 years or above. The most commonly reported domain in which difficulties were experienced were self-care (12.1%), walking/climbing (7.0%), and vision (6.3%). Hearing (2.9%), communication (3.0%), and remembering/concentrating (3.8%) were less commonly reported.

### 3.3. Prevalence of Visual Impairment

The age and sex-adjusted prevalence of blindness, severe visual impairment, and moderate visual impairment with available correction was 0.8%, 3.1%, and 8.8%, respectively. The prevalence of blindness was higher among women (1.1% compared to 0.5% among men), although was not statistically significant (Table 2). All levels of visual impairment were higher among older participants (aged over 50 years) (Table S1).

The prevalence of visual impairment at all levels was much higher among people with non-visual disabilities. Thus, the prevalence of blindness among people with non-visual disabilities was 4.5% compared to 0.3% among people without non-visual disability. Similarly, the prevalence of severe and moderate visual impairment was 9.2% and 21.1% compared to 2.3% and 7.2% among people without non-visual disabilities. The differences were statistically significant at all levels of visual impairment (Table 3). Data on household relative wealth suggested no relationship between household wealth and visual impairment.

**Figure 3:**
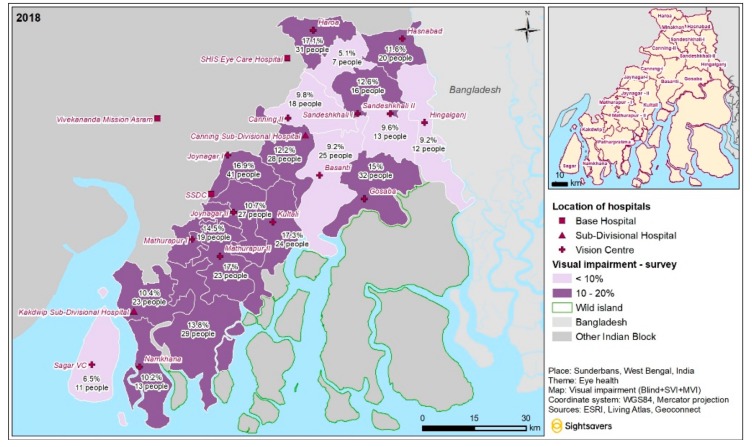
Visual impairment cases in different administrative blocks of Sunderbans, India.

Figure 3 shows the number and proportion of people with any visual impairment in different areas of Sunderbans. In six out of 19 blocks, the proportion of people with visual impairment was below 10%; in the remaining blocks, it ranged between 10% and 20%. Spatial autocorrelation showed that the likelihood of this distribution occurring by chance was very small (*p* < 0.0001).

### 3.4. Causes of Visual Impairment

The leading cause of blindness and SVI was cataract, which was responsible for 83.3% and 92.9% of cases, respectively. The main cause of MVI was refractive error (58.3%), followed by cataract (33.8%) (Table 4).

### 3.5. Cataract Coverage

Age and sex adjusted cataract surgical coverage (CSC) was 89.4% at VA 3/60, 74.1% at VA 6/60, and 61% at VA 6/18 (Table 5. Similar to the prevalence data, there was no difference in CSC by household wealth, but there were differences by disability. Thus, age and sex adjusted CSC for eyes at the 3/60 level was 76.8% among people reporting non-visual disability and 91.8% among those with no non-visual disability. Similarly, at VA 6/60 CSC for eyes was 57.3% among persons with non-visual disability and 70.1% among persons without a non-visual disability.

## 4. Discussion

Planning and implementing quality eye care services in a region with complex geography requires evidence generated for different population sub-groups who are living in the region. Population-based surveys of eye health are relatively expensive and as such are not conducted very often in many parts of India, including the Sunderbans [21,22,23].

The survey presented here shows that the prevalence of blindness among people aged over 40 years in the Indian Sunderbans is relatively low and the cataract coverage is relatively high. This is likely to be due to a well-established eye care programme with a range of eye care providers and frequent support from iNGOs. The area has a number of hospitals with eye care services, as well as a range of vision centres and government primary care centres offering primary eye care services.

Furthermore, the financial and technical support that is available to health care providers through international development projects as well as government support programmes has allowed for the delivery of eye care services at affordable prices or free of charge for certain groups of patients. It has also allowed for the provision of travel subsidies and/or transport for people in remote parts of the Sundarbans to enable them to have cataract surgery in private eye-health facilities, and the government hospitals were able to increase their surgical capacity and the number of cataract surgeries performed. This financial support and the subsidies for more vulnerable groups are likely to be the reason why we did not detect differences in visual impairment by wealth.

However, the data highlighted a number of other inequalities that still remain in the area. Similar to other studies [22,23], we found that the prevalence of blindness was higher among women (1.1% compared to 0.5%). Blindness and other types of visual impairment were also much higher among people with non-visual disabilities. This is not surprising given the difficult geography of the islands. The data highlights that women and people with additional (non-visual) difficulties do not benefit from the available services to the same extent as men or people without additional disabilities and therefore, eye health inequalities persist despite the overall success and high coverage of the available services. To address these inequalities, health care providers need to consider more proactive targeted approaches to find these hard to reach population groups and counteract the barriers that they face. Given that cataract was shown to be the main cause of both blindness and severe visual impairment and there were significant differences in CSC by disability status, new approaches are required to address this remaining burden of cataract with a particular focus on people with additional (non-visual) disabilities. GIS data that were collected in this study is useful to supplement epidemiological data and can assist in future programming and in developing strategies to reach hard to reach populations. Not only are GIS tools useful to identify underserved geographic areas, data visualisation using maps is also a powerful tool for communicating complex information to policy makers and health programme managers and supports more effective decision-making by them.

## 5. Conclusions

Data from the survey of visual impairment in the Sunderbans shows that the region is on track for achieving the goals of VISION 2020 for India. The progress is due to the investments made by the government and international development organisations in the increased human resource capacity, improved infrastructure, and diverse eye health activities across different blocks of the Sunderbans. However, there are population groups and geographic areas that find it more difficult to access the available eye health services than others. Women and people with disabilities should be given a particular priority in the regional eye care programme to facilitate the progress towards universal eye health coverage in the Sunderbans. The integration of additional tools for collecting socio-economic and GIS data in the visual impairment surveys is useful to provide evidence on the underserved populations and support more effective and targeted planning. GIS data and maps should also be used in the provision of services to support adaptive programming and direct community mobilization and outreach activities of eye care teams. Areas of higher prevalence of visual impairment should be specifically targeted with additional interventions, which may include additional travel support or awareness raising and communication campaigns. Given that one of the priority groups in the Sunderbans is people with disabilities, the involvement of locally based patient groups or Disabled People’s Organizations may be particularly beneficial to increase the uptake of services.

## Figures and Tables

**Table 1:** Socio-demographic characteristics of the survey participants.

Characteristics	2018					
	Male		Female		Total	
	n	%	n	%	N	%
Age group						
40–49	689	41.8	882	50.0	1571	46.1
50–59	443	26.9	425	24.1	868	25.5
60–69	304	18.5	280	15.9	584	17.1
70–79	161	9.8	113	6.4	274	8.0
80+	50	3.0	63	3.6	113	3.3
Mean age	54 years		52 years		53 years	
Educational Qualification						
Cannot read and write	442	26.8	842	47.8	1284	37.7
Can read/write but did not attend school	131	8.0	168	9.5	299	8.8
Primary	705	42.8	574	32.6	1279	37.5
Secondary	249	15.1	135	7.7	384	11.3
Higher secondary	53	3.2	30	1.7	83	2.4
Graduate	58	3.5	11	0.6	69	2.0
Post-graduate	9	0.6	3	0.2	12	0.4
Type of disability						
Disability	212	12.9	290	16.5	502	14.7
Non-visual disability	153	9.3	224	12.7	377	11.1

**Table 2:** Age and sex adjusted prevalence of blindness, severe visual impairment (SVI), and moderate visual impairment (MVI).

Vision Category	2018 % (95% CI)		
	Male	Female	Total
Blindness, PVA <3/60	0.5% (0.2%–0.9%)	1.1% (0.7%–1.8%)	0.8% (0.5%–1.2%)
SVI, PVA <6/60 to 3/60	3.1% (2.2%–4.3%)	3.0% (2.3%–4.0%)	3.1% (2.5%–3.8%)
MVI, PVA <6/18 to 6/60	8.4% (6.8%–10.3%)	9.3% (7.5%–11.4%)	8.8% (7.5%–10.4%)

**Table 3:** The prevalence of age and sex adjusted bilateral blindness, severe visual impairment, and visual impairment by wealth and disability.

Characteristics	Categories	VA<3/60 in the Better Eye with Available Correction	VA<6/60-3/60 in the Better Eye with Available Correction	VA<6/18-6/60 in the Better Eye with Available Correction
		% (95% CI)		
Household Wealth quintile (*p* = 0.3)	Poorest	-	-	-
	Second	0.3 (0.0–2.2)	1.4 (0.6–3.1)	6.8 (4.7–10.0)
	Third	1.0 (0.5–2.0)	3.9 (2.9–5.1)	8.9 (7.1–11.1)
	Fourth	0.8 (0.4–1.6)	2.9 (2.1–4.0)	9.6 (7.8–11.7)
	Richest	0.6 (0.2–2.5)	3.5 (2.1–5.6)	7.8 (4.7–12.5)
Disability (*p* < 0.001)	With non-visual disability	4.5 (2.7–7.2)	9.2 (6.5–12.7)	21.1 (17.3–25.5)
	Without non-visual disability	0.3 (0.2–0.6)	2.3 (1.6–3.1)	7.2 (6.0–8.7)

**Table 4:** Main causes of visual impairment.

Cause		N (%)			
		Blind	SVI	MVI	Total
Refractive Error	Total	0 (-)	1 (1.0)	169 (58.3)	170 (41.3)
	Male	0 (-)	1 (1.8)	89 (59.7)	90 (42.5)
	Female	0 (-)	0 (-)	80 (56.7)	80 (40.0)
Cataract (untreated)	Total	20 (83.3)	91 (92.9)	98 (33.8)	209 (50.7)
	Male	7 (87.5)	51 (92.7)	47 (31.5)	105 (49.5)
	Female	13 (81.3)	40 (93.0)	51 (36.2)	104 (52.0)
Corneal Scar	Total	1 (4.2)	0 (-)	2 (0.7)	3 (0.7)
	Male	0 (-)	0 (-)	2 (1.3)	2 (0.9)
	Female	1 (6.3)	0 (-)	0 (-)	1 (0.5)
Others	Total	3 (12.5)	6 (12.1)	21 (7.2)	30 (7.3)
	Male	1 (12.5)	3 (5.5)	11 (7.4)	15 (7.1)
	Female	2 (12.5)	3 (7.0)	10 (7.1)	15 (7.5)
Total	Total	24	98	290	412
	Male	8	55	149	212
	Female	16	43	141	200

**Table 5:** Age and sex adjusted cataract surgical coverage (CSC) among persons.

Categories	2018		
	Male	Female	Total
VA<3/60	93.1%	86.3%	89.4%
VA<6/60	76.3%	72.1%	74.1%
VA<6/18	61.6%	61.5%	61.0%

## Supplementary Materials

The following are available online at https://www.mdpi.com/1660-4601/16/23/4869/s1, Table S1: Age and sex adjusted prevalence of blindness, severe visual impairment (SVI), and visual impairment (VI)—all causes, 2018.
