# Supplementary material for: Integrating Geospatial Data and Measures of Disability and Wealth to Assess Inequalities in an Eye Health Survey: An Example from the Indian Sunderbans
Source: Int J Environ Res Public Health. 2019 Dec 3;16(23):4869. doi: 10.3390/ijerph16234869 (PMC6926603; doi:10.3390/ijerph16234869)
Supplement: Supplementary file 1 [file ijerph-16-04869-s001.pdf]

Table S1. Age and sex adjusted prevalence of blindness, severe visual impairment (SVI) and visual impairment (VI) – all causes, 2018

Level of visual acuity	Male			Female			Total		
	40-49	50+	All	40-49	50+	All	40-49	50+	All
	N (%; 95%CI)	N (%; 95%CI)	N (%; 95%CI)	N (%; 95%CI)	N (%; 95%CI)	N (%; 95%CI)	N (%; 95%CI)	N (%; 95%CI)	N (%; 95%CI)
**Blindness – VA<3/60 in the better eye with best correction, or pinhole**									
All bilateral blindness	-	13,242 (0.8%; 0.4-1.6%)	13,242 (0.5%; (0.2 – 0.9%)	-	27,615 (1.8%; 1.1 – 3.0%)	27,615 (1.1%; 0.6 – 1.7%)	-	40,856 (1.3%; 0.9-2.0%)	40,856 (0.7%; 0.5 – 1.1%)
All blind eyes	6,799 (0.3%; 0.1-0.7%)	82,106 (2.5%; 1.7-2.5%)	88,905 (1.6%; 1.1-2.2%)	10,493 (0.5%; 0.2-1.0%)	134,101 (4.4%; 3.2-6.1%)	144,594 (2.8%; 2.1-3.7%)	17,293 (0.4%; 0.2-0.7%)	216,207 (3.4%; 2.7-4.3%)	233,500 (2.1%; 1.7-2.7%)
**Blindness – VA<3/60 in the better eye with available correction (presenting VA)**									
All bilateral blindness	-	13,242 (0.8%; 0.4-1.6%)	13,242 (0.5%; (0.2 – 0.9%)	-	29,690 (2.0%; 1.2-3.2%)	29,690 (1.1%; 0.7-1.8%)	-	42,932 (1.4%; 0.9-2.0%)	42,932 (0.8%; 0.5-1.2%)
All blind eyes	6,799 (0.3%; 0.1-0.7%)	96,043 (2.9%; 2.1-4.0%)	102,842 (1.8%; 1.3-2.5%)	10,493 (0.5%; 0.2-1.0%)	139,657 (4.6%; 3.4-6.2%)	150,151 (2.9%; 2.2-3.8%)	17,293 (0.4%; 0.2-0.7%)	235,700 (3.7%; 3.0-4.6%)	252,993 (2.3%; 1.9-2.9%)
**Severe Visual Impairment (SVI) – VA <6/60-3/60 in better eye with available correction**									
All bilateral SVI	1,650 (0.1%; 0-1.0%)	86,270 (5.2%; 3.8-7.2%)	87,920 (3.1%; 2.2-4.3%)	1,045 (0.1%; 0-0.7%)	77,598 (5.1%; 3.9-6.8%)	78,643 (3.0%; 2.3-4.0%)	2,695 (0.1%; 0-0.5%)	163,868 (5.2%; 4.1-6.5%)	166,563 (3.1%; 2.5-3.8%)
All SVI eyes	8,648 (0.4%; 0.2-0.8%)	196,841 (6.0%; 4.6-7.8%)	205,489 (3.6%; 2.8-4.6%)	4,195 (0.2%; 0.1-0.6%)	206,622 (6.8%; 5.5-8.4%)	210,816 (4.1%; 3.3-5.0%)	12,843 (0.3%; 0.1-0.5%)	403,463 (6.4%; 5.3-7.7%)	416,306 (3.8%; 3.2-4.5%)
**Moderate Visual Impairment (MVI) - VA<6/18-6/60 in the better eye with available correction**									
All bilateral VI	23,897 (2.0%; 1.1-3.5%)	215,900 (13.1%; 10.7-15.9%)	239,797 (8.4%; 6.8-10.3%)	13,614 (1.2%; 0.7-2.3%)	227,222 (15.1%; 12.1-18.6%)	240,837 (9.3%; 7.5-11.4%)	37,512 (1.6%; 1.0-2.5%)	443,123 (14.0%; 11.9-16.5%)	480,634 (8.8%; 7.5-10.4%)
All VI eyes	77,893 (3.2%; 2.1-4.8%)	536,181 (16.3%; 13.6-19.4%)	614,073 (10.8%; 8.9-12.9%)	57,635 (2.6%; 1.8-3.9%)	538,579 (17.8%; 14.7-21.5%)	596,215 (11.5%; 9.5-13.8%)	135,528 (2.9%; 2.1-4.0%)	1,074,759 (17.0%; 14.5-19.8%)	1,210,288 (11.1%; 9.5-13.0%)
